# Steroid-Induced Thrombosis: A Comprehensive Analysis Using the FAERS Database

**DOI:** 10.3390/ph18101463

**Published:** 2025-09-28

**Authors:** Ayame Watanabe, Yoshihiro Uesawa

**Affiliations:** Department of Medical Molecular Informatics, Meiji Pharmaceutical University, Tokyo 204-8588, Japan

**Keywords:** steroids, FAERS database, venous thromboembolism, arterial thrombosis, sex hormones, pharmacovigilance

## Abstract

**Background/Objectives:** Thrombosis, a critical condition that can have severe consequences, such as myocardial infarction and cerebral infarction, can be induced by steroid drugs. Although the mechanisms for inducing thrombosis are known for some types of steroid drugs, much remains unknown about the differences in the tendency and mechanisms for thrombosis. **Methods:** To address this knowledge gap, we analyzed the relationship between thrombosis and steroid use by utilizing the U.S. Food and Drug Administration Adverse Event Reporting System database. From the database, we extracted demographic and drug information and information on reported adverse events from 2004 to 2024. We characterized drugs according to physiological function, receptor specificity, and Anatomic Therapeutic Chemical classification and calculated the proportion of steroid drugs that were likely to induce thrombosis. **Results:** Among steroid drugs, sex hormones such as androgens, progestogens, and estrogens appeared to have particularly high potential for causing thrombotic events. Results of principal component analysis and cluster analysis indicated that sex hormone preparations were associated with an increased risk of venous thrombosis. In addition, cardiovascular medications and mineralocorticoids, which are used to treat diseases of major organs, showed a tendency to induce large-vessel occlusions. **Conclusions:** These findings may be useful for selecting steroid drugs for patients who are at risk for similar adverse effects.

## 1. Introduction

Because of their anti-inflammatory and immunosuppressive effects, steroid drugs are used widely in the treatment of various diseases [1,2]. Pharmaceuticals with a steroid backbone can be categorized into several types, including glucocorticoids, mineralocorticoids, sex hormones, and anabolic steroids [3]. Glucocorticoids have immunosuppressive and anti-inflammatory properties and are used to treat nearly all autoimmune diseases, chronic inflammatory disorders, allergies, and some malignant tumors [4]. Sex hormones are broadly categorized as female and male hormones, and female hormones are subdivided into progestins and estrogens. These are used primarily for contraception and osteoporosis prevention and in the treatment of menstrual disorders, menopausal symptoms, and certain cancers [5,6,7,8,9,10]. Anabolic steroids generally promote muscle growth and protein synthesis; they are used medicinally to treat muscle weakness caused by various conditions [11,12].

However, steroids are also known to cause various adverse effects [13]. A population-based cohort study in the United States reported that within 30 days after initiating short-term oral corticosteroids, the incidence rate ratio (IRR) for sepsis was 5.30; for venous thromboembolism, 3.33; and for fractures, 1.87 [13]. However, the mechanisms underlying these adverse effects, including thrombosis, remain largely unclear. In Japan, the Ministry of Health, Labor, and Welfare has issued guidelines addressing thrombosis as a serious adverse effect, which underscores the need for further research [14].

Thrombosis can be broadly classified into arterial and venous thrombosis [15]. A shared underlying mechanism is endothelial dysfunction. Under normal conditions, endothelial cells produce nitric oxide and prostacyclin, which suppress platelet aggregation, promote fibrinolysis via tissue plasminogen activator, and inhibit coagulation via anticoagulant factors. In endothelial dysfunction, these protective functions are lost, which leads to a thrombogenic state. Dysfunctional endothelium increases lipoprotein permeability, causing accumulation in the subendothelial space and triggering local inflammation, macrophage differentiation, and foam cell formation. These processes eventually lead to the development of atherosclerotic plaque and arterial thrombosis. In atherosclerotic regions, the anticoagulant activity of endothelial cells is reduced, which accelerates the development of thrombosis. Conversely, venous thrombosis is mainly caused by blood stasis and hypercoagulability. Factors such as inflammation, hypoxia, and mechanical stress caused by blood pooling may induce endothelial dysfunction, in which tissue factor is expressed, which initiates the coagulation cascade. Hypoxia from venous stasis activates thrombogenic factors, such as plasminogen activator inhibitor-1 and von Willebrand factor, while suppressing fibrinolysis, which exacerbates endothelial dysfunction. This vicious cycle characterizes venous thrombosis [16,17]. Although these mechanisms are largely elucidated, the tendency of different types of steroid drugs to induce specific types of thrombosis remains unclear [18]. We hypothesized that the tendency to develop thrombosis (e.g., arterial vs. venous) varies by steroid class.

The U.S. Food and Drug Administration (FDA) Adverse Event Reporting System (FAERS) [19] is a collection of numerous reports of adverse events from the United States and abroad, which is useful for analyzing the relationship between thrombosis and steroid drugs. The present study aimed to identify steroid drugs associated with thrombotic events using the FAERS database and characterize their tendencies and features, such as whether they are more likely to induce arterial or venous thrombosis, based on their pharmacological classification.

## 2. Results

### 2.1. Creation of the Data Table

**Figure 1:**
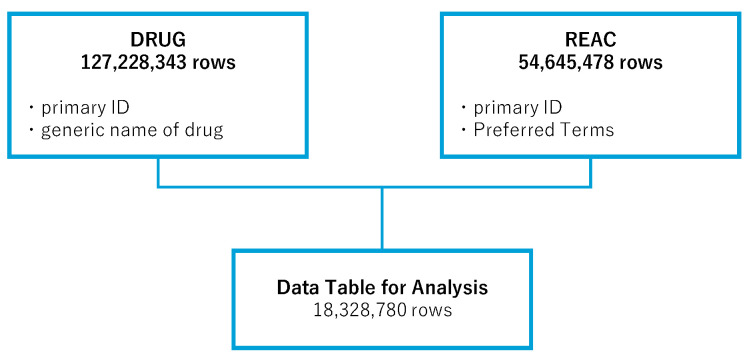
Flowchart of the process for creating the analysis data table. Data in the DRUG (drug information) and REAC (adverse event information) tables from the FDA Adverse Event Reporting System (FAERS) were combined, allowing for duplicates, and were linked by means of the primary ID number.

In the analysis, the FAERS drug information (DRUG) table (127,228,343 rows) and the adverse event (REAC) table (54,645,478 rows) were used. We combined the information in these tables to create an integrated data table, consisting of 18,328,780 rows, for analysis. Duplicate entries were removed during the creation of the data table (Figure 1).

### 2.2. Steroid Drugs That Induce Thrombosis

**Figure 2:**
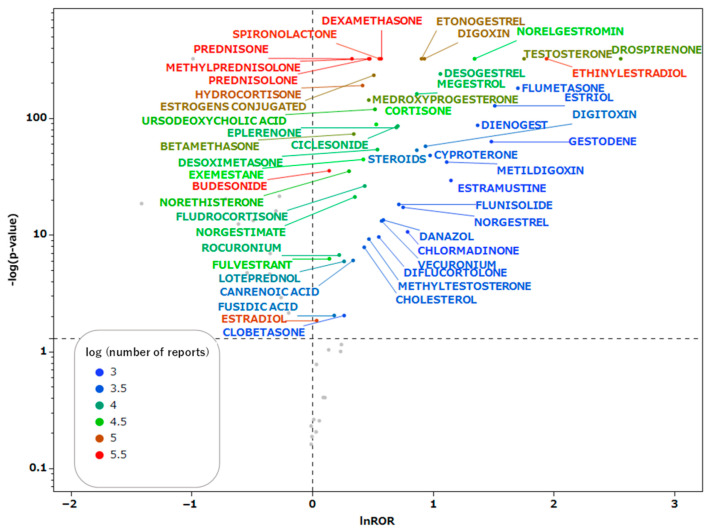
Volcano plot for steroids and thromboses. The vertical axis represents the statistical significance according to Fisher’s exact test, and the horizontal axis represents the risk of inducing thromboses. The steroid names and their leaders are colored according to the number of reports.

All steroid drugs included in the integrated data table are listed in Table 1. The FDA approval status of the target drugs was investigated using DailyMed, and the 71 approved drugs have been listed in Supplementary Table S3. In addition, 90 types of steroids with adverse event reports since 2024 were identified, as listed in Supplementary Table S4. These drugs were confirmed to be still in use clinically. In the present study, all steroids, including these aforementioned drugs, were analyzed. Scatter plots were generated for drugs with 1000 or more reported adverse events in the data table. These scatter plots (Figure 2) depict the correlation between steroid drugs and thrombosis. Each plot represents a steroid drug; the greater the natural logarithm of the reporting odds ratio (ROR) and the negative logarithm of the *p*-value [−log(*p* value)] (Supplementary Table S1), the more statistically significant the drug is estimated to induce thrombosis. Steroid drugs for which the natural logarithm of the ROR > 0 and *p* < 0.05 were documented and analyzed.

### 2.3. Relationship Between Patient Age and Sex and Thrombosis Induced by Steroids

Table 2 presents data on the sex and age of patients who used steroids. The results revealed that the risk of thrombosis was higher in female patients than in male patients. Although the age difference was statistically significant, the medians were nearly identical, suggesting that age-related differences were minimal.

### 2.4. Relationship Between Administration Route and Thrombosis Induced by Steroids

Table 3 presents data on the association between various routes of steroid administration and the risk of thrombosis. The results revealed that the oral, topical, transdermal, and vaginal routes were significantly (all *p* < 0.001) associated with an increased risk of thrombosis. Conversely, the respiratory (inhalation), intrauterine, subdermal, and nasal routes were significantly associated with a decreased risk of thrombosis.

### 2.5. Classification of Steroid Drugs and Their Relationships to Thrombogenesis

Steroids with a high potential to induce thrombosis (lnROR > 0, *p* < 0.05) were categorized according to their physiological functions, receptor specificity, and Anatomical Therapeutic Chemical (ATC) classification. We then calculated the proportions of steroids in each classification (Table 4). According to the results of Fisher’s exact test, androgens significantly increased the risk of thrombosis (ROR = 5.305, *p* < 0.05).

### 2.6. Principal Component Analysis

**Figure 3:**
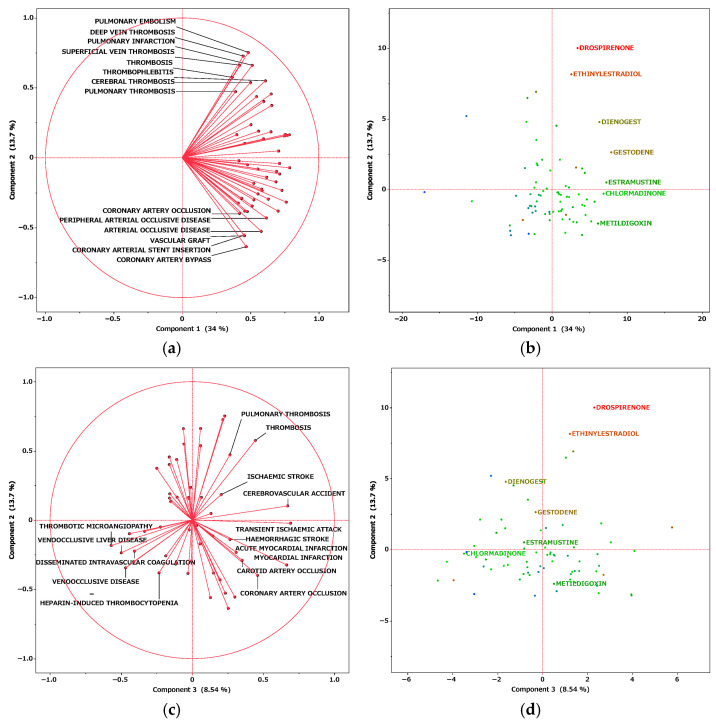
Relationships of thromboses with steroids according to principal components analysis. (**a**,**b**) Results for components 1 (physiological functions) and 2 (receptor specificity). (**c**,**d**) Results for components 2 and 3 (Anatomic Therapeutic Chemical classification). Loading plots (**a**,**c**) represent the association between adverse events related to thromboses and each principal component. Each loading vector represents an adverse effect. Score plots (**b**,**d**) represent the relationship between steroids and each principal component. Each dot refers to a specific steroid drug.

**Figure 4:**
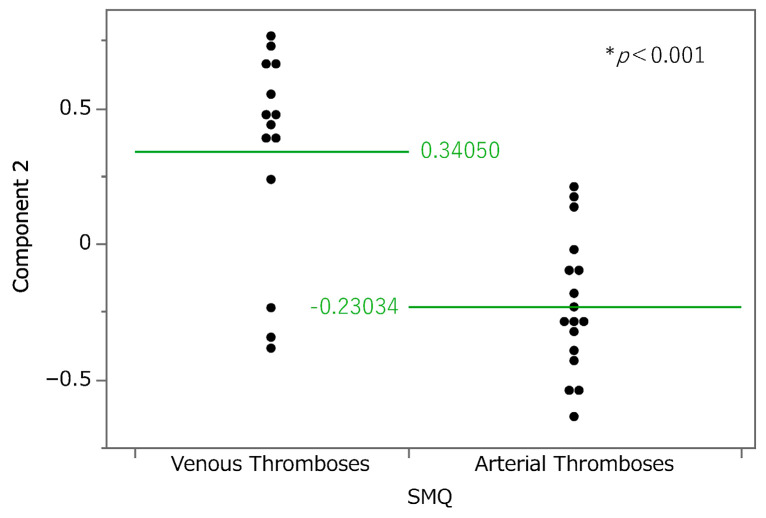
The relationship between component 2 and each steroid at the level of the Standardised Medical Dictionary for Regulatory Activities (MedDRA) Query (SMQ). Cases classified as “Thromboses of unspecified or mixed vessels” were excluded. The average values of principal component 2 for each SMQ were 0.3050 for “Venous embolism and thrombosis” and −0.23034 for “Arterial embolism and thrombosis.” * Welch’s *t* test was performed to assess the statistical significance of the difference.

We performed a principal component analysis (Figure 3). According to the results, the contribution rates of principal components 1, 2, and 3 were 34.0%, 13.7%, and 8.54%, respectively. Component 1 is considered to be associated with the estimated risk of thrombosis onset because all the loading vectors of thrombosis-related terms were in a positive direction. For component 2, pulmonary embolism, deep vein thrombosis, and superficial vein thrombosis had high positive loading vectors. Adverse events whose vectors loaded highly in a negative direction included coronary artery bypass, arterial occlusive disease, and peripheral arterial occlusive disease. Thus, higher values of principal component 2 were associated with a tendency toward venous thrombosis. The relationship between principal component 2 and thrombosis-related terms was analyzed at the Standardised Medical Dictionary for Regulatory Activities (MedDRA) Query (SMQ) level (Figure 4); SMQs are systematically grouped MedDRA terms related to specific medical areas of interest and are used to comprehensively and efficiently extract and analyze terms for safety evaluations and adverse event analyses. Drugs classified under the SMQ for “arterial embolism and thrombosis” had low values for principal component 2, whereas those under “venous embolism and thrombosis” had high values. With regard to principal component 3, loading vectors in the positive direction included those for transient ischemic attack, stroke, myocardial infarction, hemorrhagic stroke, and coronary artery occlusion. In contrast, loading vectors in the negative direction included those for disseminated intravascular coagulation, thrombotic microangiopathy, and heparin-induced thrombocytopenia.

### 2.7. Hierarchical Cluster Analysis

**Figure 5:**
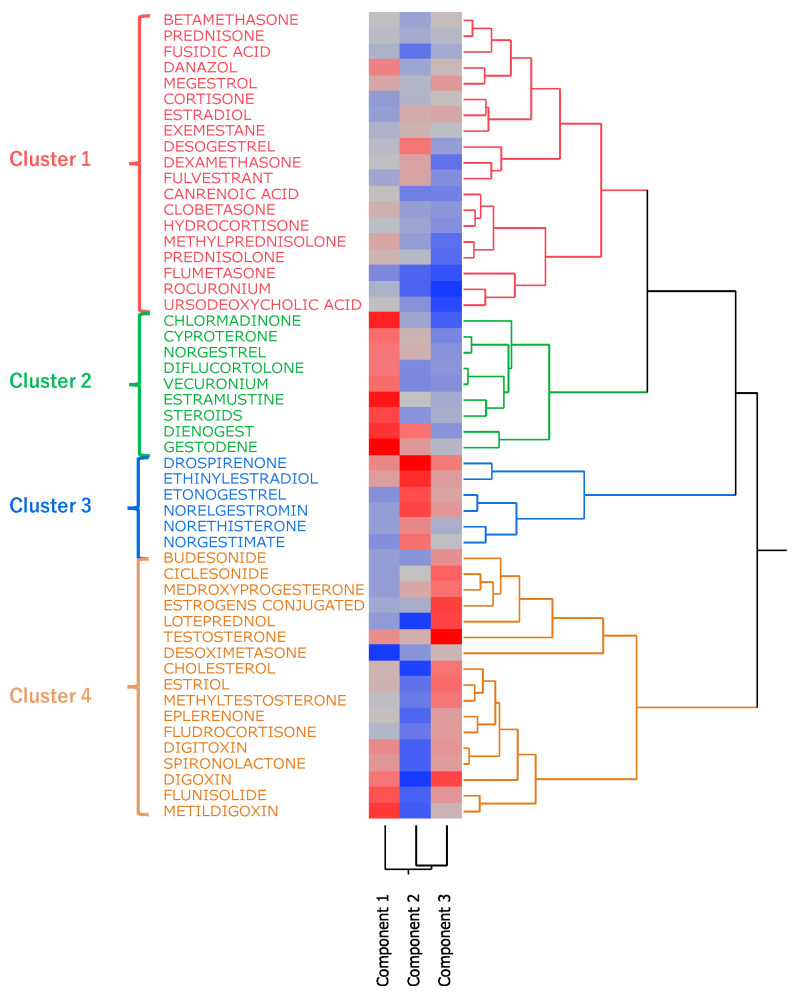
Results of hierarchical cluster analysis, depicting the relationship between 52 side effects related to thromboses and 51 steroids. In the color map, the redder the color, the higher the value of each principal component, and the bluer the color, the lower the value.

**Figure 6:**
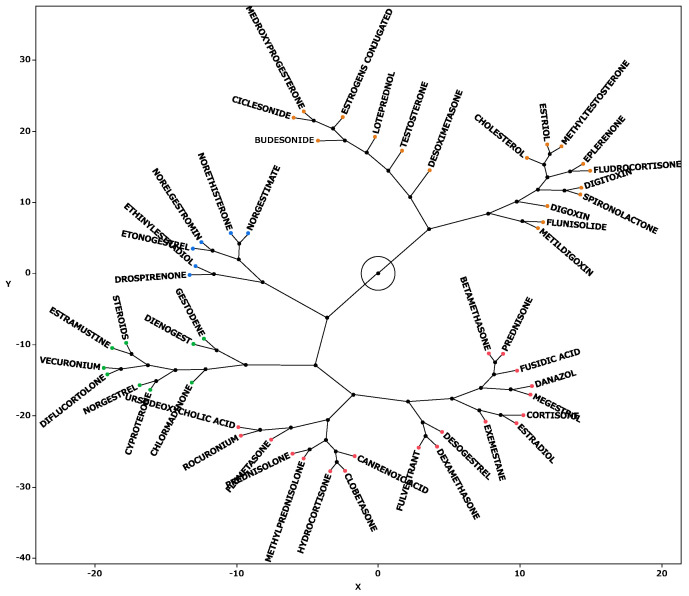
Constellation dendrogram. The figure enables a visual understanding of the results of the cluster analysis. Dots indicate individual data lines, and line lengths indicate relative distances to clusters.

Hierarchical cluster analysis performed with principal components 1, 2, and 3 (see Section 2.6) resulted in the classification of drugs into four broad clusters (Figure 5). Drugs with high values for principal component 1 included sex hormone preparations found across clusters, as well as digoxin, digitoxin, and methyldigoxin in cluster 4. Cluster 3 was characterized by drugs with high values for principal component 2, whereas drugs with high values for principal component 3 were included primarily in cluster 4. On the basis of the results of the cluster analysis, we created a constellation dendrogram to visualize the relationship between drugs and adverse events (Figure 6).

## 3. Discussion

### 3.1. Characteristics of Patients with Thrombosis and Steroids

Age and sex are important modulators of thrombosis risk associated with the use of corticosteroids and hormonal agents. In the present study, although the difference in age between the groups was statistically significant, the medians were nearly identical, indicating that age-related differences were minimal (Table 2). However, previous studies have reported that aging itself is a risk factor for thrombosis [13,20,21]. Further research is warranted to clarify this association. In our analysis, the ROR for female steroid users was significantly elevated at 1.201 (95% confidence interval = 1.176–1.225). Previous epidemiological studies also identified an elevated risk of venous thromboembolism among women using oral contraceptives; however, no direct comparison of odds ratios between the sexes has been performed [22]. Although the association between aging and thrombotic risk is well documented in the literature, direct evaluation of sex-based differences in steroid-associated thrombosis remains limited, warranting further investigation in future research.

### 3.2. Impact of the Route of Steroid Administration on Thrombotic Risk

The oral, topical, transdermal, and intravaginal routes of administration significantly increased the risk of thrombosis (Table 3). Previous studies have reported that oral estrogen preparations are associated with a significantly higher risk of venous thromboembolism than transdermal formulations [23,24]. This elevated risk is attributable to the hepatic first-pass effect, during which the synthesis of procoagulant factors is enhanced and anticoagulant factors are suppressed [23,24,25]. Furthermore, oral administration results in a rapid postprandial increase in serum hormone concentrations, inducing a transient prothrombotic state, whereas nonoral routes are associated with a more gradual increase in hormone levels.

Conversely, the present analysis revealed that administration routes that bypass the hepatic first-pass effect, such as the topical, transdermal, and intravaginal routes, were also associated with an increased risk of thrombosis. Steroid hormones with a high thrombogenic potential, including androgens, estrogens, and progestins, are commonly administered orally, transdermally, or intravaginally. In menopausal hormone therapy, both estrogen monotherapy and estrogen–progestin combination therapy are typically administered orally, transdermally, or intravaginally [26]. Moreover, a claims database study conducted in Japanese postmenopausal women between 2005 and 2021 demonstrated that the predominant form of estrogen prescription was transdermal estradiol and that the use of oral dydrogesterone and transdermal norethisterone acetate increased over time [27].

Furthermore, progesterone preparations are widely used orally and intravaginally for endometrial protection during estrogen combination therapy and for luteal phase support in assisted reproductive technology, with intravaginal administration also reported to be effective in preventing preterm birth [28]. FDA-approved administration routes for testosterone replacement therapy include buccal, intranasal, subcutaneous, transdermal, and intramuscular delivery, with transdermal formulations being widely used in clinical practice [29].

Overall, in addition to the inherently high thrombotic risk of these agents, the high RORs observed for certain routes, particularly the transdermal, topical, and intravaginal routes, which bypass the first-pass effect, might be attributable to their frequent use for the administration of highly thrombogenic steroid hormones. Notably, the occurrence of adverse events associated with steroid therapy is strongly influenced by both the dosage and duration of administration [30]. As these factors were not considered in the present analysis, further research is warranted to more precisely clarify the relationship between steroid hormone administration routes and thrombotic risk.

### 3.3. Classification of Steroid Drugs with a High Thrombogenic Potential

In this study, 51 steroid drugs showed statistically significant associations with thrombosis (those for which the natural logarithm of the ROR > 0 and *p* < 0.05). These 51 drugs were categorized into 15 therapeutic groups according to the ATC classification. Of these groups, the androgen class exhibited the highest thrombogenic potential (ROR = 5.305, *p* < 0.001), followed by progestogens (ROR = 3.572) and estrogens (ROR = 3.227). These results suggest that sex hormone preparations, in particular, have a strong tendency to induce thrombosis. Previous studies have also confirmed an increased risk of venous thromboembolism with testosterone therapy, attributed to enhanced thrombogenesis and reduced fibrinolysis [31]. Moreover, results from the large Dutch MEGA case–control study showed an increased risk of venous thromboembolism with oral contraceptives, with risk varying by estrogen dose and progestogen type [22]. Our findings are consistent with this previous evidence; thus, clinicians must carefully consider the risk of thrombosis when administering steroid drugs related to sex hormones, and preventive measures and monitoring must be implemented when necessary. In particular, considering the discussion in Section 2.3 (Table 2), caution is warranted regarding the use of hormone preparations in women. Unlike previous studies that focused on specific steroid drugs, the novelty of our research lies in our use of the large-scale FAERS database, which enabled a comprehensive analysis of the entire spectrum of steroid drugs. This approach allowed us to identify associations between thrombosis and steroid drugs that had not been sufficiently studied before, such as cardiac glycosides (e.g., digoxin) and anabolic steroids.

### 3.4. Principal Component Analysis

Principal component analysis is a technique used to reduce the dimensionality of a dataset, enhancing interpretability while minimizing information loss [32]. In this study, principal component 1 represented the estimated risk of thrombosis onset; principal component 2 represented the type of thrombosis, with a positive correlation with venous thrombosis and a negative correlation with arterial thrombosis. This finding suggests that the vectors for drugs associated with venous thrombosis are loaded in the positive direction, whereas those associated with arterial thrombosis are loaded in the negative direction. Principal component 3 appears to correspond to occlusive events in major vessels of vital organs in the positive direction and to microvascular-level thrombosis in the negative direction. On the basis of this hypothesis, score plots were examined. Drugs strongly associated with principal component 1, and thus with high potential for inducing thrombosis in general, included gestodene, estramustine, and chlormadinone. Drugs such as drospirenone, ethinylestradiol, and norelgestromin were strongly and positively associated with principal component 2, which indicated a high potential for inducing venous thrombosis. In addition, loteprednol, digoxin, and obeticholic acid were estimated to have high potential for inducing arterial thrombosis. A strong association with principal component 3, which characterized testosterone, conjugated estrogens, and digoxin, suggested a potential to cause occlusion of major vessels in vital organs. Previous studies have also indicated that digoxin and testosterone are associated with cardiac events and an increased risk of arteriosclerosis [33,34,35]. Conversely, drugs such as rocuronium, ursodeoxycholic acid, and flumethasone were estimated to be more likely to induce microvascular thrombosis. These findings are expected to be useful in monitoring thrombosis according to the type of steroid drug administered to patients.

### 3.5. Cluster Analysis

Hierarchical cluster analysis is a method used to group and classify similar data [36]. As a result of our analysis, the drugs were classified into four clusters (Figure 5 and Figure 6), and the drugs included in each cluster are listed in Table 5. Cluster 2 contained drugs with high values for principal component 1. All drugs in cluster 3 and some in other clusters had high values for principal component 2. These drugs included drospirenone, ethinylestradiol, and etonogestrel, most of which were sex hormone preparations, particularly progestogens or estrogens. This suggests that female hormone preparations have potential for inducing venous thrombosis. Cluster 4 showed a strong correlation with principal component 3 and included drugs such as testosterone, digoxin, and loteprednol [29,33,34,35,37,38]. These drugs are used in the treatment of diseases in major organs and include cardiovascular medications [33,34,35].

### 3.6. Study Limitations

This study had several limitations. First, the FAERS database contains reports of spontaneous adverse events; it does not provide information about all patients who were administered the drugs, and thus, the true incidence of adverse events cannot be calculated, and absolute risk assessments cannot be performed. Furthermore, spontaneous reports of adverse events are subject to reporting bias, including underreporting, overreporting, and misreporting. This bias could have affected our analyses of FAERS data. Second, some values may be missing from the FAERS database, and some included reports may be inaccurate. To address this, we excluded data suspected to be missing or erroneous in the age and gender data tables. Third, the number of drugs analyzed was limited by the number of reported cases. Information not considered in this analysis—such as patients’ underlying diseases, concomitant drug use (presence, type, and number), and methods and duration of drug administration—could have affected the manifestation of adverse events. In particular, when multiple drugs are administered, determining which drug caused the adverse event may be difficult [39]. Furthermore, it should be acknowledged that adverse drug reactions, including steroid-associated thrombotic events, may arise from a constellation of contributing factors such as genetic variability, epigenetic influences, pharmacodynamic and pharmacokinetic properties, and potential drug–drug interactions. These underlying factors were not considered in this analysis, but they might have influenced the observed reporting patterns. The observed associations do not indicate causality. Disproportionality analyses, such as those using the FAERS database, are hypothesis-generating tools, and they cannot establish a direct cause–effect relationship between steroid use and thrombosis. Further research is expected to yield insights that take these confounding factors into account.

## 4. Materials and Methods

### 4.1. FAERS Database

FAERS is a large-scale database consisting of case reports. The FAERS comprises seven data tables: DEMO, which contains patient information such as age, sex, and weight; DRUG, which includes drug information; REAC, which contains information on reported adverse events; OUTC, which contains descriptions of clinical outcomes; RPSR, which provides the information sources; INDI, which contains data on drug indications; and THER, which includes details about therapy dates and treatment progress. In this analysis, the DRUG and REAC tables were integrated on the basis of a unique identifier. This allowed for the creation of a unified dataset (the integrated table described in Section 2.1) in which each record corresponded to a specific drug and adverse event. To avoid overestimation in case-based aggregation, duplicate cases based on the same unique identifier were excluded. To accurately assess the effects and adverse events of individual drugs, we excluded records of cases involving combination therapies, focusing only on groups in which the target drug was used alone. For this study, we utilized FAERS data from the first quarter of 2004 (January–March) through the third quarter of 2024 (July–September). Because the data were open access and anonymized, this study was exempt from ethical review and informed consent by the Meiji Pharmaceutical University Ethics Committee.

### 4.2. Selection of Target Drugs and Control of Adverse Events

To obtain Simplified Molecular Input Line Entry System (SMILES) representations of drug names listed in FAERS, we used the Python library PubChemPy (https://github.com/mcs07/pubchempy, accessed on 13 June 2025) [40] to search the PubChem database. Of the 5523 drugs in FAERS that were assigned SMILES representations, 233 drugs containing a steroid backbone were extracted and analyzed (Table 4). The following SMILES arbitrary target specification expression for the steroid backbone was used in the analysis:

“[#6]~1~[#6]~[#6]~2~[#6]~[#6]~[#6]~3~[#6](~[#6]~[#6]~[#6]~4~[#6]~[#6]~[#6]~[#6]~[#6]~3~4)~[#6]~2~[#6]~1”

For extraction, the molecular operating environment [41] we used was a comprehensive computational chemistry system equipped with a scientific vector language developed by Chemical Computing Group, Montreal, Quebec, Canada. A market survey of 233 steroid drugs was conducted using two approaches. First, the FDA approval status of these drugs was investigated using DailyMed [42]. Second, the FAERS drug and therapy tables were combined to identify steroid drugs for which the first administration occurred in 2024 or later; this indicated that the drug in question was currently in clinical use. For adverse event analysis, we applied the SMQ [43] from MedDRA version 27.1 [44]. For analysis, we utilized 419 narrow-scope preferred terms from the SMQs for “Arterial embolism and thrombosis,” “Venous embolism and thrombosis,” and “Embolism and thrombosis of unspecified or mixed vessels.” Of these, 399 preferred terms were found in the FAERS database.

### 4.3. Calculation of RORs

The ROR is a disproportionality measure used in pharmacovigilance to detect signals of potential associations between drugs and adverse events. It is based on the general concept of the odds ratio, which compares the odds of an outcome occurring in an exposed group versus a non-exposed group. In this context, the ROR compares the odds of a specific adverse event being reported for a target drug with the odds of the same event being reported for all other drugs. An ROR of 1 indicates no difference; values greater than 1 suggest a higher reporting frequency of the event with the drug of interest, and values less than 1 suggest a lower frequency. To calculate the ROR, we constructed 2 × 2 contingency tables (drug vs. adverse event; Table 6). When any cell contained zero, we applied a continuity correction by adding 0.5 to all cells (Haldane’s correction) to avoid infinite estimates [45,46]. The ROR was then calculated as [(a/b)/(c/d) = (a × d)/(b × c)], where a is the number of reports of the event with the drug, b the number of reports without the event for the drug, c the number of reports of the event with all other drugs, and d the number of reports without the event for all other drugs.

### 4.4. Creation of Scatter Plots

From the 2 × 2 contingency tables described in Section 4.3, the RORs and *p*-values from Fisher’s exact test were calculated. Because some drug–event combinations had small numbers of reports, we applied Fisher’s exact test, a statistical method that computes an exact *p*-value for a 2 × 2 table without relying on large-sample approximations. This approach ensures reliable results even when counts are low. A *p*-value of less than 0.05 was considered statistically significant, meaning that the observed difference in reporting was unlikely to be due to chance. The ROR for each drug represents the strength of association with the adverse event, thrombosis, and the *p*-value indicates statistical significance. We created a volcano plot in which the vertical axis represented the negative common logarithm of the *p*-value (−log *p*) and the horizontal axis represented the natural logarithm of the ROR [47,48,49,50,51]. Drugs for which the natural logarithm of the ROR > 0 and *p* < 0.05 [47,48,49,50,51] and for which 1000 or more adverse events were reported, as well as 52 thrombosis-related preferred terms included in 2000 or more reports of adverse events, were used in the subsequent cluster and principal component analyses. The drugs and preferred terms included in the analysis are summarized in Table 7 and Table 8.

### 4.5. Classification of Steroid Drugs with High Potential for Inducing Thrombosis

The analyzed drugs were classified into 18 categories based on physiological function, receptor specificity, and ATC classification (Table 9). We calculated the proportion of steroid drugs within each category that were likely to induce thrombosis.

### 4.6. Principal Component Analysis

We performed principal component analysis for the drugs listed in Table 7 (Supplementary Table S2). We then used the results, focusing on principal components 1, 2, and 3, to create association diagrams.

### 4.7. Hierarchical Cluster Analysis

In a method similar to that for the principal component analysis, we used the aggregated table from Section 4.4 and principal components 1, 2, and 3 for the cluster analysis. The Ward method [36,52] was employed as the clustering technique.

### 4.8. Statistical Analysis

To merge the data tables, we used Python (version 3.12.7). To perform statistical analyses, we used JMP Pro 18 (SAS Institute Inc., Cary, NC, USA). A *p*-value of less than 0.05 was considered statistically significant.

## 5. Conclusions

This study identified associations between 51 steroid drugs and thrombosis as signals of disproportionate reporting in the FAERS database. This analysis revealed distinct patterns differentiating the risks of venous and arterial thrombosis among these drugs, providing new evidence to inform risk assessment in steroid therapy. However, as a retrospective signal detection study, our results are hypothesis-generating and should be interpreted with caution. Future research, including observational studies or randomized controlled trials, is necessary to validate these associations. In patients with established risk factors for thrombosis, the indication for steroid use should be carefully reassessed. Therapeutic strategies that avoid the use of steroids are warranted, particularly when alternative treatments are available. Furthermore, a detailed investigation into receptor-mediated mechanisms may enable the development of drugs that do not activate thrombogenic pathways. Based on such insights, future efforts should focus on the creation of safer therapeutic options. Clinicians and regulatory scientists should remain vigilant by considering these potential risks when prescribing steroid medications. In particular, for patients with a high predisposition to thrombosis, prophylactic measures and careful monitoring should be implemented as appropriate. In addition, mechanistic investigations into thrombosis-related pathways are crucial for elucidating the biological basis of these thrombotic effects, which could guide the development of safer and more effective therapeutic alternatives.

## Figures and Tables

**Table 1:** Drugs that have a steroid structure.

EPRISTERIDE	MEPROSCILLARIN	DIFLUPREDNATE	ORYZANOL
BOLDENONE	PENGITOXIN	FLUCLOROLONE ACETONIDE	GUGGULSTERONE
CHLORODEHYDROMETHYLTESTOSTERONE	STROPHANTHIN-K	FLUDROXYCORTIDE	WITHANIA SOMNIFERA
CLOSTEBOL	ACETYLDIGITOXIN	FLUMETASONE	BETA-SITOSTEROL
DROSTANOLONE	ACETYLDIGOXIN	FLUOCINOLONE ACETONIDE	BRASSICASTEROL
MESTANOLONE	DESLANOSIDE	FLUOCINONIDE	CAMPESTEROL
METHANDRIOL	DIGITOXIN	FLUOCORTIN	SITOSTEROLS
METHASTERONE	DIGOXIN	FLUOROMETHOLONE	STIGMASTANOL
METRIBOLONE	LANATOSIDE C	FLUPREDNIDENE	STIGMASTEROL
OXYMESTERONE	METILDIGOXIN	FLUTICASONE	EMERGENCY CONTRACEPTIVES
TRENBOLONE	OUABAIN	HALCINONIDE	ALGESTONE
ETHYLESTRENOL	PROSCILLARIDIN	HALOMETASONE	DIMETHISTERONE
METANDIENONE	IODINE (131 I) NORCHOLESTEROL	METHYLPREDNISOLONE	GESTODENE
METENOLONE	VAMOROLONE	MOMETASONE	NORELGESTROMIN
NANDROLONE	20-HYDROXYECDYSONE	PREDNICARBATE	NORETYNODREL
NORETHANDROLONE	COLLAGENASE	TRIAMCINOLONE	NORGESTIMATE
OXYMETHOLONE	EQUILIN	ULOBETASOL	NORGESTREL
QUINBOLONE	ESTETROL	CLOPREDNOL	SEGESTERONE
STANOZOLOL	ESTROGENS	CORTISONE	TRIMEGESTONE
FLUOXYMESTERONE	MESTRANOL	CORTIVAZOL	ALLYLESTRENOL
NORMETHANDRONE	METHYLESTRADIOL	DEFLAZACORT	CHLORMADINONE
ANDROSTANOLONE	QUINESTRADOL	FLUOCORTOLONE	DESOGESTREL
DANAZOL	QUINESTROL	HYDROCORTISONE	DIENOGEST
MESTEROLONE	EPIMESTROL	MEPREDNISONE	DROSPIRENONE
METHYLTESTOSTERONE	ESTRADIOL	PARAMETHASONE	DYDROGESTERONE
PRASTERONE	ESTRIOL	PREDNISOLONE	ETHISTERONE
TESTOSTERONE	ESTROGENS CONJUGATED	PREDNISONE	ETONOGESTREL
7-KETO-DEHYDROEPIANDROSTERONE	ESTRONE	PREDNYLIDENE	ETYNODIOL
ANDROSTENEDIOL	ESTROPIPATE	RIMEXOLONE	GESTONORONE
ANDROSTENEDIONE	ETHINYLESTRADIOL	PREDNIMUSTINE	HYDROXYPROGESTERONE
ANECORTAVE	MOXESTROL	FLUMEDROXONE	LEVONORGESTREL
GALETERONE	PROMESTRIENE	CICLESONIDE	LYNESTRENOL
MEPITIOSTANE	2-METHOXYESTRADIOL	FLUNISOLIDE	MEDROGESTONE
CLASCOTERONE	ESTRAMUSTINE	TIXOCORTOL	MEDROXYPROGESTERONE
CYPROTERONE	CIPROCINONIDE	FORMOCORTAL	MEGESTROL
ABIRATERONE	DEPRODONE	LOTEPREDNOL	NOMEGESTROL
FULVESTRANT	DEXBUDESONIDE	MEDRYSONE	NORETHISTERONE
ONAPRISTONE	ETIPREDNOL DICLOACETATE	ORIC 101	NORGESTRIENONE
TELAPRISTONE	FLUOCORTIN BUTYL	ALDOSTERONE	PROGESTERONE
GESTRINONE	FLUPAMESONE	DESOXYCORTONE	PROMEGESTONE
MIFEPRISTONE	FLUPREDNISOLONE	FLUDROCORTISONE	TIBOLONE
DEHYDROCHOLIC ACID	HALOPREDONE	CANRENOIC ACID	PREGNANDIOL
HYODEOXYCHOLIC ACID	MAZIPREDONE	CANRENONE	GINSENOSIDE RG3
NORUCHOLIC ACID	PREDNAZOLINE	EPLERENONE	ULIPRISTAL
CHENODEOXYCHOLIC ACID	PREDNISOLAMATE	SPIRONOLACTONE	STEROIDS
CHOLIC ACID	PROCINONIDE	ALFADOLONE	FUSIDIC ACID
OBETICHOLIC ACID	ALCLOMETASONE	ALORADINE	ATAMESTANE
URSODEOXYCHOLIC ACID	AMCINONIDE	MINAXOLONE	EXEMESTANE
DEOXYCHOLIC ACID	BECLOMETASONE	PREGNENOLONE	FORMESTANE
SODIUM TAUROCHOLATE	BETAMETHASONE	ZURANOLONE	RUSCOGENIN
TAUROSELCHOLIC ACID	BUDESONIDE	ALFAXALONE	TRILOSTANE
URSODOXICOLTAURINE	CLOBETASOL	GANAXOLONE	CHOLESTEROL
BETA-ACETYLDIGOXIN	CLOBETASONE	BREXANOLONE	EPICHOLESTANOL
CINOBUFAGIN	CLOCORTOLONE	RAPACURONIUM BROMIDE	NORCHOLESTENOL IODOMETHYL
CINOBUFOTALIN	DESONIDE	PANCURONIUM	SELENONORCHOLESTEROL (75 SE)
CONVALLATOXIN	DESOXIMETASONE	PIPECURONIUM	CHOLESTERYL BENZOATE
DIGITALIN	DEXAMETHASONE	ROCURONIUM	VITAMIN D1
GITALOXIN	DIFLORASONE	VECURONIUM	
LANATOSIDES	DIFLUCORTOLONE		

**Table 2:** Sex and age of patients who received steroids.

		Thrombosis	Non-Thrombosis	ROR	95%Cl	*p*-Value
sex	Female	18,120 (3.56%)	338,603 (55.3%)	1.201	1.176−1.225	<0.001
	Male	21,806 (2.96%)	234,331 (38.2%)			
age (median)		61.46	59.9			<0.001

**Table 3:** Reporting odds ratios for thrombosis induction by route of administration.

Route	Thrombosis	Non-Thrombosis	ROR	95%CI	*p*-Value
ORAL	39,238	457,022	1.3723	1.3563–1.3885	<0.0001
RESPIRATORY (INHALATION)	6420	165,336	0.5659	0.5517–0.5805	<0.0001
INTRA-UTERINE	1227	134,234	0.1308	0.1236–0.1384	<0.0001
INTRAVENOUS	7282	92,632	1.1865	1.1580–1.2158	<0.0001
TOPICAL	7271	61,065	1.8200	1.7753–1.8657	<0.0001
TRANSDERMAL	4959	40,806	1.8465	1.7921–1.9025	<0.0001
VAGINAL	5508	26,604	3.1739	3.0818–3.2687	<0.0001
SUBDERMAL	475	38,715	0.1816	0.1659–0.1989	<0.0001
NASAL	1758	35,644	0.7368	0.7021–0.7731	<0.0001
INTRAMUSCULAR	1952	29,763	0.9831	0.9389–1.0295	0.4768

**Table 4:** Distribution on volcano plots and the *p*-values and RORs by drug class.

Steroid Classification	Steroids Likely to Induce Thromboses *#	Number of All Steroids by Drug Class *	Steroids Likely to Induce Thromboses/Number of All Steroids by Steroid Class (%)	ROR	95%Cl	*p*-Value
Androgen	3	4	75.0	5.305	5.203−5.408	<0.0001
Antiandrogen	1	2	50.0	2.651	2.363−2.973	<0.0001
Antiestrogen	1	1	100.0	1.151	1.090−1.214	<0.0001
Antiprogestogen	0	1	0.0	−	−	−
Bile Acid	1	3	33.3	1.679	1.612−1.748	<0.0001
Cardiac Glycoside	3	3	100.0	2.532	2.476−2.589	<0.0001
Enzyme	0	1	0.0	−	−	−
Estrogen	5	7	71.4	3.327	3.292−3.360	<0.0001
Glucocorticoid	15	27	55.6	1.510	1.500−1.519	<0.0001
Mineralocorticoid	1	1	100.0	1.543	1.432−1.661	<0.0001
Mineralocorticoid Receptor Antagonist	3	3	100.0	1.746	1.713−1.780	<0.0001
Non-steroidal Neuromuscular Blocker	2	2	100.0	1.353	1.261−1.450	<0.0001
Phytosteroid	0	1	0.0	−	−	−
Progestogen	12	17	70.6	3.572	3.533−3.610	<0.0001
Steroid (General)	1	1	100.0	2.375	2.153−2.618	<0.0001
Steroidal Antibiotic	1	1	100.0	1.198	1.051−1.365	0.0092
Steroidal Aromatase Inhibitor	1	1	100.0	1.525	1.442−1.611	<0.0001
Sterol	1	1	100.0	1.536	1.336−1.765	<0.0001

* Steroids likely to induce thrombosis as indicated by *p* < 0.05 and ROR > 1 by Fisher’s exact test. # These numbers represent the counts of various steroids.

**Table 5:** Drug clusters.

Cluster 1		Cluster 2		Cluster 3		Cluster 4	
DANAZOL	Androgen	CYPROTERONE	Antiandrogen	ETHINYLESTRADIOL	Estrogen	METHYLTESTOSTERONE	Androgen
FULVESTRANT	Antiestrogen	ESTRAMUSTINE	Estrogen	NORELGESTROMIN	Progestogen	TESTOSTERONE	
URSODEOXYCHOLIC ACID	Bile Acid	DIFLUCORTOLONE	Glucocorticoid	NORGESTIMATE		DIGITOXIN	Cardiac Glycoside
ESTRADIOL	Estrogen	VECURONIUM	Non-steroidal Neuromuscular Blocker	DROSPIRENONE		DIGOXIN	
BETAMETHASONE	Glucocorticoid	GESTODENE	Progestogen	ETONOGESTREL		METILDIGOXIN	
CLOBETASONE		NORGESTREL		NORETHISTERONE		ESTRIOL	Estrogen
DEXAMETHASONE		CHLORMADINONE				ESTROGENS CONJUGATED	
FLUMETASONE		DIENOGEST				BUDESONIDE	Glucocorticoid
METHYLPREDNISOLONE		STEROIDS	Steroid (General)			DESOXIMETASONE	
CORTISONE						CICLESONIDE	
HYDROCORTISONE						FLUNISOLIDE	
PREDNISOLONE						LOTEPREDNOL	
PREDNISONE						FLUDROCORTISONE	Mineralocorticoid
CANRENOIC ACID	Mineralocorticoid Receptor Antagonist					EPLERENONE	Mineralocorticoid Receptor Antagonist
ROCURONIUM	Non-steroidal Neuromuscular Blocker					SPIRONOLACTONE	
DESOGESTREL	Progestogen					MEDROXYPROGESTERONE	Progestogen
MEGESTROL						CHOLESTEROL	Sterol
FUSIDIC ACID	Steroidal Antibioteic						
EXEMESTANE	Steroidal Aromatase Inhibitor						

**Table 6:** Cross-tabulation of the reporting odds ratios.

	Thromboses	Non-Thromboses Disorders
Reports with the suspected drug	a	b
All other reports	c	d

Reporting odds ratio (ROR) = [(a/b)/(c/d) = (a × d)/(b × c)].

**Table 7:** Drugs for analysis.

ABIRATERONE	DEXAMETHASONE	FLUMETASONE	MOMETASONE
BECLOMETASONE	DIENOGEST	FLUNISOLIDE	NORELGESTROMIN
BETAMETHASONE	DIFLUCORTOLONE	FLUOCINOLONE ACETONIDE	NORETHISTERONE
BUDESONIDE	DIFLUPREDNATE	FLUOCINONIDE	NORGESTIMATE
CANRENOIC ACID	DIGITOXIN	FLUOROMETHOLONE	NORGESTREL
CHLORMADINONE	DIGOXIN	FLUTICASONE	OBETICHOLIC ACID
CHOLESTEROL	DROSPIRENONE	FULVESTRANT	PRASTERONE
CICLESONIDE	DYDROGESTERONE	FUSIDIC ACID	PREDNISOLONE
CLOBETASOL	EPLERENONE	GESTODENE	PREDNISONE
CLOBETASONE	ESTRADIOL	HYDROCORTISONE	PROGESTERONE
COLLAGENASE	ESTRAMUSTINE	HYDROXYPROGESTERONE	ROCURONIUM
CORTISONE	ESTRIOL	LEVONORGESTREL	SPIRONOLACTONE
CYPROTERONE	ESTROGENS	LOTEPREDNOL	STEROIDS
DANAZOL	ESTROGENS CONJUGATED	MEDROXYPROGESTERONE	TESTOSTERONE
DEFLAZACORT	ESTROPIPATE	MEGESTROL	TIBOLONE
DEOXYCHOLIC ACID	ETHINYLESTRADIOL	METHYLPREDNISOLONE	TRIAMCINOLONE
DESOGESTREL	ETONOGESTREL	METHYLTESTOSTERONE	ULOBETASOL
DESONIDE	EXEMESTANE	METILDIGOXIN	URSODEOXYCHOLIC ACID
DESOXIMETASONE	FLUDROCORTISONE	MIFEPRISTONE	VECURONIUM
			WITHANIA SOMNIFERA

**Table 8:** Preferred terms for analysis.

PT		
ACUTE CORONARY SYNDROME	EMBOLIC STROKE	PULMONARY INFARCTION
ACUTE MYOCARDIAL INFARCTION	EMBOLISM	PULMONARY THROMBOSIS
ARTERIAL OCCLUSIVE DISEASE	EMBOLISM VENOUS	RETINAL ARTERY OCCLUSION
BLINDNESS TRANSIENT	HAEMORRHAGIC STROKE	RETINAL VEIN OCCLUSION
CARDIAC VENTRICULAR THROMBOSIS	HEMIPARESIS	STRESS CARDIOMYOPATHY
CAROTID ARTERY OCCLUSION	HEMIPLEGIA	SUPERFICIAL VEIN THROMBOSIS
CENTRAL VENOUS CATHETERISATION	HEPARIN-INDUCED THROMBOCYTOPENIA	THROMBOPHLEBITIS
CEREBRAL INFARCTION	INFARCTION	THROMBOSIS
CEREBRAL ISCHAEMIA	ISCHAEMIC STROKE	THROMBOSIS IN DEVICE
CEREBRAL THROMBOSIS	LACUNAR INFARCTION	THROMBOTIC MICROANGIOPATHY
CEREBROVASCULAR ACCIDENT	MONOPLEGIA	THROMBOTIC THROMBOCYTOPENIC PURPURA
CEREBROVASCULAR DISORDER	MYOCARDIAL INFARCTION	TRANSIENT ISCHAEMIC ATTACK
CORONARY ARTERIAL STENT INSERTION	PARAPLEGIA	VASCULAR GRAFT
CORONARY ARTERY BYPASS	PARESIS	VENOOCCLUSIVE DISEASE
CORONARY ARTERY OCCLUSION	PERIPHERAL ARTERIAL OCCLUSIVE DISEASE	VENOOCCLUSIVE LIVER DISEASE
DEEP VEIN THROMBOSIS	PORTAL VEIN THROMBOSIS	VENOUS THROMBOSIS
DEVICE OCCLUSION	PULMONARY EMBOLISM	VENOUS THROMBOSIS LIMB
DISSEMINATED INTRAVASCULAR COAGULATION		

**Table 9:** Classification of steroids.

Steroid Classification	
Androgen	Mineralocorticoid
Antiandrogen	Mineralocorticoid Receptor Antagonist
Antiestrogen	Non-steroidal Neuromuscular Blocker
Antiprogestogen	Phytosteroid
Bile Acid	Progestogen
Cardiac Glycoside	Steroid (General)
Enzyme	Steroidal Antibiotic
Estrogen	Steroidal Aromatase Inhibitor
Glucocorticoid	Sterol

## Data Availability

Data are contained within the article and Supplementary Materials.

## Supplementary Materials

The following supporting information can be downloaded at https://www.mdpi.com/article/10.3390/ph18101463/s1. Table S1: List of 77 steroids with potential risk of inducing thromboses based on RORs and *p*-values; Table S2: lnROR matrix for steroids and thromboses; Table S3: FDA approval status of steroid drugs; Table S4: Steroid drugs with adverse event reports since 2024.
